# Deep learning in cancer genomics and histopathology

**DOI:** 10.1186/s13073-024-01315-6

**Published:** 2024-03-27

**Authors:** Michaela Unger, Jakob Nikolas Kather

**Affiliations:** 1 Else Kroener Fresenius Center for Digital Health, Medical Faculty Carl Gustav Carus, TUD Dresden University of Technology, Dresden, Germany; 2 Department of Medicine I, University Hospital Dresden, Dresden, Germany; 3 Medical Oncology, National Center for Tumor Diseases (NCT), University Hospital Heidelberg, Heidelberg, Germany

**Keywords:** Deep learning, Precision oncology, Histopathology, Genomics, Multimodality

## Abstract

Histopathology and genomic profiling are cornerstones of precision oncology and are routinely obtained for patients with cancer. Traditionally, histopathology slides are manually reviewed by highly trained pathologists. Genomic data, on the other hand, is evaluated by engineered computational pipelines. In both applications, the advent of modern artificial intelligence methods, specifically machine learning (ML) and deep learning (DL), have opened up a fundamentally new way of extracting actionable insights from raw data, which could augment and potentially replace some aspects of traditional evaluation workflows. In this review, we summarize current and emerging applications of DL in histopathology and genomics, including basic diagnostic as well as advanced prognostic tasks. Based on a growing body of evidence, we suggest that DL could be the groundwork for a new kind of workflow in oncology and cancer research. However, we also point out that DL models can have biases and other flaws that users in healthcare and research need to know about, and we propose ways to address them.

## Background

Precision oncology is based on diagnostic histopathological and genomic methods, which enable the application of a suitable therapy to patients [1]. Histopathology investigates the morphology, or phenotype, of a tumor and is indispensable to diagnose and subtype cancer. One of the most general and widely used methods in histopathology is staining of tissue slides with hematoxylin and eosin (H&E) [2]. To complement the phenotypic information, genomic biomarkers are routinely used for patients with advanced or metastatic cancer since they exhibit a predictive power for the patient’s survival or for the effectiveness of a cancer drug. Thus, in many cases, genomics allows a more personalized form of therapy [3]. Given these advancements, it is not surprising that precision oncology could improve clinical outcomes in the last decades [4, 5]. However, precision oncology is inherently data-intensive: to support treatment decisions, a wide range of data is required, including general patient information such as age, biological sex, medical history, patient preferences, radiological imaging, histopathology, and molecular and genetic assays. At the same time, the amount of available information beyond patient data is extensive as well. For example, in 2021, the US Food and Drug Administration (FDA) had approved a total of 243 cancer drugs for patient therapy [6]. Combined, the quantity of patient-specific data and the number of treatment options create a vast decision tree which is becoming more complex to navigate for patients and physicians. Therefore, there is a need for tools to support cancer care by efficiently utilizing and analyzing all available information.

One solution for this growing demand could be the application of computer-aided methods. Improvements in computer hardware and algorithms have multiplied our abilities to process large-scale data since the late 20th century. Today, artificial intelligence (AI) methods have become ubiquitous tools in our everyday life. AI can solve complex tasks at the level of human experts, such as in language translation and object detection [7, 8]. This is also true for biomedical research, where AI is able to solve complex problems like predicting protein folding from amino acid sequences [9] or analyzing and interpreting radiology imaging data [10]. As a potential advantage over human skills, AI methods are scalable and can process vast amounts of data in a relatively short time.

**Fig. 1:**
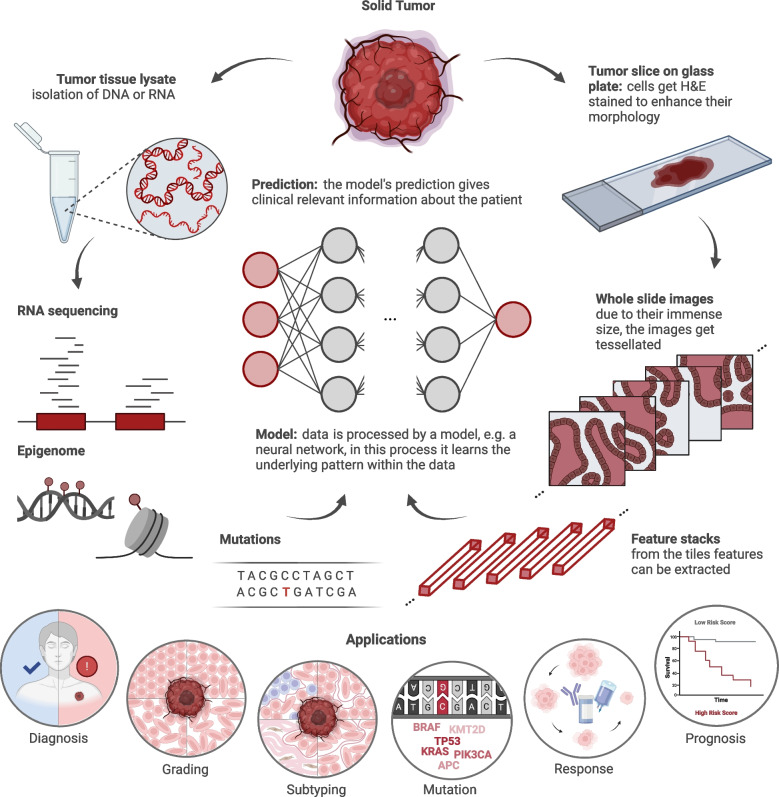
Workflow of AI in histopathology and clinical genomics. In this simplified workflow, a tissue of a solid tumor is harvested via surgery or biopsy. One part is sequenced in the genomics facility to obtain molecular data about, for instance, RNA, epigenetics, or mutations, while another part is sent to the pathology department. There, tumor slices are captured on glass slides and stained with hematoxylin and eosin (H&E). Images of these glass slides can then be taken. Tabular and image data are used to train models, e.g., neural networks to provide a prediction. In this review, we describe six distinct medical application tasks (Diagnosis, Grading, Subtyping, Mutation, Response, and Survival) for these models

One most fundamental component of AI is machine learning (ML). There are three main approaches to ML: reinforcement, unsupervised, supervised learning. In reinforcement learning, the model is rewarded for making correct decisions. In unsupervised learning, the model is tasked to learn from data, but is given no additional information about it. For example, clustering methods can identify similar instances in a given dataset, without being provided with explicit labels on each instance. Supervised learning, in contrast, can use human-labeled data and tasks the model with automating the labeling process. A portion of this data is given to the model to predict labels, and the model is penalized when it gives the wrong output. Model architectures used for supervised learning include support vector machines (SVMs), decision trees and artificial neural networks. These models can vary greatly in size, with the number of parameters ranging from hundreds of parameters to billions of parameters in neural networks [11]. Whenever ML is applied to image or text data, deep artificial neural networks, also known as deep learning (DL) [12], are the favored models due to their robustness and effectiveness in handling complex data structures. In precision oncology, AI with DL can process large amounts of histopathologic and genomic data (Fig. 1) [1, 13, 14]. Notably, some studies even adopted multimodal models that apply ML and DL to several data types simultaneously, such as combining histopathological images with genetic data [15–17]. This approach of multimodal data integration could potentially improve model performance by incorporating additional patient information and leveraging synergistic effects between complementary data types.

Here, we provide a high-level overview of DL’s role in pathology, genomics, and multimodal data analysis. To bring structure to the diversity in the academic literature, we establish a guiding framework. In our analysis, we divide our investigation into six fields of clinically focused application, as established by previous studies [18]. Three “basic” applications are as follows: predicting the diagnosis (cancer detection), subtype, and grading of a tumor; and three “advanced” applications are as follows: predicting prognosis (survival probability of the patient), patterns of genetic alterations (such as the detection of driver mutations), or treatment response to a specific treatment scheme or a single medicine [18–20]. Furthermore, we discuss the potential limitations of DL approaches in clinical routines and provide insights into future trajectories of these fields. Altogether, this review should not only inform about the most recent developments in the area but also inspire researchers to further contribute to this topic and close its existing gaps.

## DL in histopathology

Histopathology is a fundamental part of precision oncology. Virtually all solid tumor entities must be diagnosed by histopathology or cytology. In essence, all clinical decisions based on treatment and follow-up depend on histopathological information. In digital pathology, tissue slides are digitally captured as whole slide images (WSI) in high resolution, yielding images with billions of pixels, or “gigapixel images.” AI can process such digital information and has emerged as the default tool to automate diagnostic processes and identify new biomarkers in WSIs (Fig. 1).

Most AI studies in histopathology employ supervised DL. Of particular relevance are “weakly” supervised approaches, in which the objective of the system is to predict a “label” for the WSI in its entirety [13, 21, 22]. A “label” can refer to any of the basic and advanced categories, including properties of slides (presence of tumor), properties of tumors (subtype or genetic alterations), and of patients (survival or response) [13]. During training, a weakly supervised tumor detection system only has access to a label on a slide level. For example, the label could denote: “does this slide contain a tumor, yes or no?”. An alternative approach is “strongly” supervised learning. Here, the objective is to delineate tumor tissue or detect cell types based on accurate, manual annotations. Weakly supervised approaches obviate the need for manual annotation and, hence, are more scalable to large image archives. In addition, weakly supervised approaches allow us to predict more abstract properties of tumors, such as the presence of mutations or the survival of patients [13, 22–25].

### DL for basic histopathological tasks

**Fig. 2:**
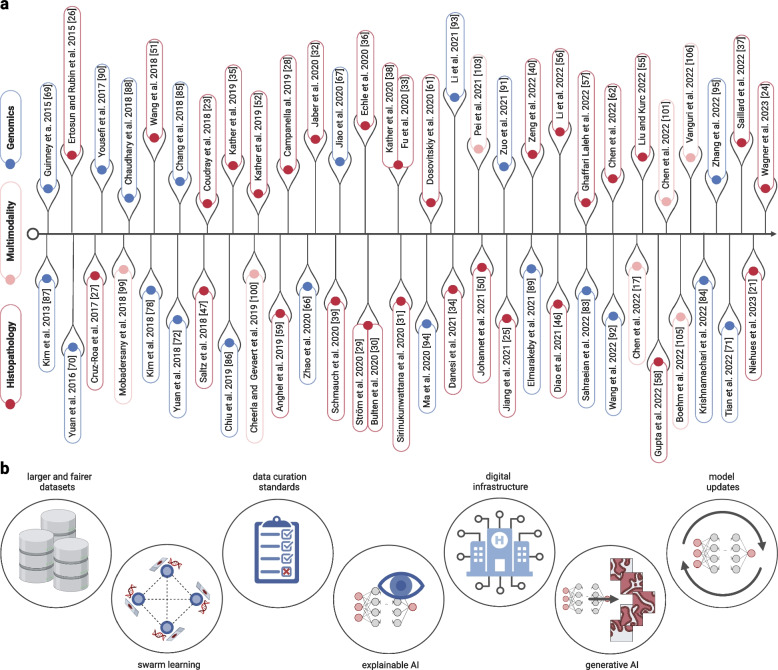
Timeline and outlook. **a** The timeline of milestone papers mentioned in this review. Articles are colored by research area (blue — genomics, rose — multimodal, red — histopathology). **b** Future perspectives AI will face in the next years to be applied in clinical routines

One of the earliest studies on weakly supervised DL in histopathology was conducted by Ertosun and Rubin in 2015 (Fig. 2a) [26], in which the authors automated histological grading in primary brain tumors using a convolutional neural network (CNN). CNNs are a type of neural network commonly used in image analysis, containing so-called convolutional layers. Vividly speaking, layers of convolution find basic structures like corners and edges in the original image which are then concatenated by the neural network to higher hierarchies, and with this, determine global patterns shared between images. Ertosun and Rubin were among the earliest to move from handcrafted features with simple ML classifiers to DL. This enabled them to address a clinically relevant classification task in computational pathology.

Prior to tumor grading or any other step, the diagnosis must take place. Hence, diagnosis is one of the most obvious and most common applications of DL in histopathology. In this task, models need to differentiate tumor tissue and healthy tissue on WSIs in a strongly or weakly supervised manner. One of the first studies which employed DL for tumor detection was carried out by Cruz-Roa et al. [27] (Fig. 2a) in 2017. The authors diagnosed breast cancer by using a CNN which was trained on almost 400 WSIs. Their model reached a high performance for tumor detection. At this time, essential preprocessing steps were already established, e.g. making large WSIs usable by tesselating them (Fig. 1). In 2019, the field of cancer detection with weakly supervised DL was markedly changed as a result of a large-scale seminal work by Campanella et al. [28] (Fig. 2a), whose multiple-instance learning model outperformed strongly supervised models with an area under the receiver operating characteristic (AUROC) curve as high as 0.986. DL models could therefore probably assist pathologists in the future by pre-labeling samples, potentially reducing the load of confirmatory molecular assays.

One year later, Ström et al. [29] and Bulten et al. [30] (Fig. 2a) demonstrated that DL was able to solve a subtyping task in solid tumors, another important application of DL. Their approaches did not only include tumor segmentation, but also prediction of Gleason grade in prostate cancer with weakly supervised learning. Complementary to these diagnostic tasks, the most influential recent study in digital pathology was published by Coudray et al. [23] (Fig. 2a) in 2018. Coudray et al. established weakly-supervised methods for the slide-level prediction of histological subtype of non-small-cell lung cancer and, importantly, showed that genetic alterations in targetable genes are predictable from histopathology slides [23]. Although straightforward in hindsight, these studies were the first large-scale evidence that weakly supervised DL could differentiate between morphologies of cancer subtypes and link the cancer genotype from morphology alone. In the subsequent years, many studies extended this methodology to other subtypes of solid tumors. A notable example is the Consensus Molecular Subtypes (CMS) of colorectal cancer, which were shown to be predictable from routine pathology slides by Sirinukunwattana et al. [31] (Fig. 2a) in 2021. Similarly, in breast cancer, Jaber et al. [32] (Fig. 2a) presented a model that classified the five molecular subtypes of breast cancer (luminal A, luminal B, *HER2*-enriched, basal-like, normal-like) from histopathology slides with high accuracy. All these studies indicate that DL could potentially streamline diagnostic workflows by automating basic diagnostic processes, like subtyping and grading. Additionally, in a broader sense, these studies show that the ground truth for DL-based predictions can be obtained from any source as long as there is a phenotypic change the model can detect.

### DL for advanced histopathological tasks

Of similar importance to the DL method that is used, is the data a model is trained on. One of the largest studies in recent years was conducted by Fu et al. [33] (Fig. 2a) incorporating more than 17,000 WSIs from the TCGA. Important to note is that the performance of DL models is dependent on the size and quality of the input. Therefore, it was not surprising that such an immense dataset led to an AUROC of 0.98 when distinguishing cancer types. However, not only did they classify cancer tissues, but they also predicted genome duplications, driver mutations like *TP53* or *BRAF*, and tumor-infiltrating lymphocyte (TIL) scores, setting the stage for a broad application of AI in creating pathology biomarkers. Genetic alterations in cancer, as predicted by Fu et al., can be drug targets, biomarkers, or both. For example, the presence of certain *BRAF* mutations in many tumor types is a direct target for treatment with *BRAF* inhibitors. A concrete biomarker is microsatellite instability (MSI), which acts as a biomarker for immune checkpoint inhibitors [34]. Some of these targets and biomarkers can be predicted with DL from pathology slides. In 2019 Kather et al. [35] (Fig. 2a) were able to predict MSI in colorectal, gastric, and endometrial cancers. As a following publication, Echle et al. [36] (Fig. 2a) trained models to predict MSI in colorectal cancer, along with the driver mutations *BRAF* and *KRAS*, in larger patient cohorts. Today, some of these approaches have been implemented by commercial entities and are being marketed as algorithms for routine clinical use in Europe [37]. In addition to predicting single gene mutations or molecular subtypes, several studies have shown that it is also possible to extract expression levels of individual genes, or panel expression profiles directly from WSIs [38–40]. Consequently, AI could in principle be used to pre-screen for a wide range of molecular alterations and suggest which targets should be further analyzed.

Another alternative for receiving information about the patient status is investigating the tumor microenvironment. The interactions between the patient’s immune system and the cancer can be relevant for overall survival [41, 42] or therapy response [43]. For example, patient outcomes can be predicted by the number of TILs [44]. Moreover, the importance of spatial biology was already known as early as 2006; however, it has not been translated to clinical routines yet [45]. On this account, DL models emerged that detect TILs and catalog cell types [46, 47] in a specimen annotation-free and in an end-to-end approach. Therefore, DL could offer an easier path to clinical application of still unused knowledge.

As mentioned before, the prediction of genomic or morphologic biomarkers from routine histology slides is clinically relevant for the patient. However, biomarkers are just proxies for clinical outcomes—survival or treatment response. Direct prediction of treatment response to specific drugs from histopathology images could theoretically even outperform the predictive power of genomic biomarkers. Thus, drug response prediction is one of the latest advanced applications in digital pathology. In 2020, a study on predicting the response to chemotherapy in nasopharyngeal cancer was published by Liu et al. [48] (Fig. 2a). Similarly, Li et al. [49] (Fig. 2a) trained a DL model to predict a pathological complete response after neoadjuvant chemotherapy. Furthermore, immunotherapy, as another form of cancer treatment, was under investigation by Johannet et al. [50] (Fig. 2a) in 2021. The fact that DL captures underlying connections between tissue morphology and treatment response shows that the predictive capabilities of such models reach far beyond human expertise. However, these studies need many comparable cases and treatment data with a consecutive target score which is why drug response is one of the most difficult applications to establish a large dataset with good quality ground truth. Therefore, the current state of DL in treatment response suggests that direct predictions require more extensive studies in the future.

The second clinical endpoint being directly predicted by DL in histopathology is the prognosis of cancer patients, i.e., forecasting patient survival. To elucidate the prognosis of a patient is from fundamental interest since therapy decisions and patient care are directly dependent on it. In DL research, early publications used, for example, shape and boundary [51] or tissue proportions [52] of tumors as features that can be linked to patient outcomes. Today, DL models construct predictive risk scores in a straightforward manner. Information about absolute survival times is collected and combined with the censoring data of each patient. Afterwards, the model can learn which pattern to connect with a longer or shorter lifespan of a patient [53, 54]. The success of this application type could also lay in its potential to reveal yet unknown relationships between survival and phenotype.

Similarly to clinical targets getting more refined over years of research, model architectures changed as well. For most early studies, CNNs were applied as the model of choice. Later, feature extraction, a process in which pretrained DL models reduce the dimensionality of input images to smaller matrices or vectors, became the state-of-the-art method [25, 55–59] (Fig 1). Another change in model design was introduced after 2017, in which transformer neural networks [60, 61] were developed. These models can weigh parts of their input differently based on an attention mechanism and parallelize the processing of multiple parts of the input data in a computationally efficient way. In 2022, Chen et al. [62] (Fig. 2a) predicted survival through the use of vision transformers, which were able to outperform convolution-based models in many cancer types.

In summary, during the last years, AI in pathology underwent many changes and trends. Starting with simple diagnostic tools the field was soon able to outperform trained pathologists in tumor detection. Subsequently, research demonstrated that patterns in WSIs can be used for prognostic tasks as well, facilitating therapy decisions based on mutational status, drug response, or overall survival. Nevertheless, rapid changes in the model landscape of DL make it challenging for companies to develop these technologies into static products. To put this into perspective, in 2023, only four AI-based tools were FDA-approved and applied in pathology [63]. Therefore, it would be clearly desirable to increase this number and move more DL tools into diagnostic routine in precision oncology.

## DL in clinical genomics

Unique molecular characteristics of a tumor are encoded in its genome [64]. Thus, research in clinical genomics is a key to delivering precision oncology since it studies the human genome with a focus on a disease genotype. Thereby, genotypic properties such as genomic instability or mutation status of the tumor complement the phenotypic and spatial changes addressed in histopathology. Clinical genomics not only employs classical genomic data from whole genome or exome sequencing, but also RNA-sequencing, methylation assays, copy number variation analyses, and more as information sources (Fig. 1). With this, it supports the identification of the patient’s exact type of cancer, its potential primary site, responsiveness to certain drugs, or the patient’s prognosis.

Previously, analyzing genomic data was only conducted by classical bioinformatics, which employed algorithms to perform tasks such as sequence alignment, variant calling, or differential expression analysis. However, these algorithms are highly hand-engineered and focus on finding patterns which are predefined by human experts. The potential utility of AI for clinical genomics is to expand this toolkit by offering the possibility of deeper data analysis than previously attainable. Patterns that are unknown or undetectable to humans, such as the way a protein folds into its final shape or the signature left by a mutagenic process in our DNA, were discovered through the use of ML [9, 65]. Revealing novel paradigms with AI could contribute to innovations in clinical genomics that are otherwise not possible for standard bioinformatics approaches.

### DL for basic genomic tasks

DL applications in genomics have developed differently than those in histopathology. Usually, genomic information is extracted after a cancer has been diagnosed and followed up histologically. As a result, DL in clinical genomics is more involved in the advanced tasks, e.g., finding biomarkers for certain therapies or drug-response, rather than streamlining workflows by diagnosing cancer. Nevertheless, DL can be utilized in patient cases where the diagnosis is not straightforward. For example, in 2020, Zaoh et al. [66] (Fig. 2a) used a DL model to predict the original tumor tissue for patients with cancer of unknown primary from RNA-sequencing data. Similarly, in the same year, Jiao et al. [67] (Fig. 2a) found that DL can be used on passenger mutation patterns to distinguish primary from metastatic tumors. Even though these studies are not focused on cancer detection, they can provide valuable insights for the downstream decision-making process.

One basic DL application that is more prominent for clinical genomics is subtyping. Articles such as Sienkiewicz et al. [68] (Fig. 2a) utilized classical unsupervised ML in the form of non-negative matrix factorization to cluster omics data of cancer patients to discover molecular subtypes. In order to refine these classes, more sophisticated models such as random forests or DL can also be employed [69–71]. DeepGene, a model developed by Yuan et al. [70] (Fig. 2a) in 2016, used somatic mutations as their information source, whereas two years later, they published another study performing the same task, this time with copy number alterations and chromatin structure data [72]. Despite these advancements, the state-of-the-art to detect major cancer subtypes remains the morphological evaluation in most cases, with some exceptions being the recently introduced classifications of brain tumors. High costs and standardization issues associated with sequencing are limitations that prevent molecular subtypes from clinical adoption [73]. Furthermore, while some molecular subtypes such as the CMS in colorectal cancer can partially be correlated to relevant clinical outcomes, a more extensive data exploration and validation is needed to provide clinical evidence and hence foster a broader acceptance in the community.

### DL for advanced genomic tasks

The task of mutation prediction from genomic data might seem contradictory, since detecting driver mutations from it forms the ground truth for DL predictions. Classical variant calling algorithms spot nucleotide changes in the cancer genome compared to a reference, with additional tools subsequently determining if the respective mutation affects a cancer-driving gene [74–77]. In these tasks, employing DL is not a necessity. Therefore, the approaches towards mutation prediction with DL differ between those for histopathology and genomics. One example for this paradigm shift is the DL-supported discovery of gene mutations previously unrelated to cancer. In 2018, Kim et al. [78] (Fig. 2a) used what are known as skip-gram networks to visualize mutations and discover novel cancer drivers. Mutations in genes such as *CRLF2*, *TFE3*, or *DUSP22* were positive hits of their method but were previously not described as driver mutations in literature. Nevertheless, to make this knowledge clinically actionable, wet lab validation studies are needed to elucidate their mechanism of action. Besides conventional driver mutations, the whole mutational spectrum of a cancer genome, including general somatic mutations, can additionally provide important insights [79, 80]. Furthermore, variant calling must be performed as a baseline to detect driver mutations. Today, there are different bioinformatic tools that process whole genome or exome sequencing data to first align reads to a reference genome and then find changes in the donor sample compared to the reference [81, 82]. Due to the complexity of this problem, research also developed DL-based methods to improve variant calling. For example, in 2022 Sahraeian et al. [83] (Fig. 2a) used CNNs to process matched tumor and normal reads to catalog somatic mutations. A similar approach was used by Krishnamachari et al. [84] (Fig. 2a) three years later. Both methods displayed superior accuracy compared to conventional bioinformatic tools. Nevertheless, the large amount of training data and high computing power needed for DL could hinder its broad adoption. Despite these challenges, our examples demonstrate that DL has the potential to detect genomic variations at diverse scales with promising results.

Drug response predictions in clinical genomics often rely on data generated via cancer cell line cultures rather than solid tumors. In pharmacogenomics, genome-wide association studies enable the simultaneous screening of a broad number of cancer-drug pairs and therefore build the foundation for many DL applications. In 2018, Chang et al. [85] (Fig. 2a) predicted drug efficacy from genomic information of cancer cell lines and drug structural information, whereas Chiu et al. [86] (Fig. 2a) relied on mutation and expression data, without incorporating information about the drug’s chemical properties. This contrasts computational pathology since cell line-based approaches are massive simplifications of human tumors. Cancer cell lines are often genetically altered to achieve immortality introducing genotypic and phenotypic biases which eventually make them less biologically comparable to primary cancer cells. Moreover, drug screens conducted in cell lines contain no other representative elements of their original tumor microenvironment. As a result, DL approaches to evaluate drug-cancer interactions come into question and call for more practical data sources.

In contrast to current genomic drug response models, DL approaches for prognosis predictions could offer a more direct integration into clinical workflows. One of the first publications regarding DL in clinical genomics predicted cancer outcomes of ovarian cancer from DNA methylation, miRNA and bulk-RNA expression, and copy number alterations (CNAs). The software package ATHENA, developed by Kim et al. [87] (Fig. 2a), incorporated this data into grammatical evolution neural networks. Here, over several iterations, sets of neural networks with varying parameters are constructed, and the best-performing networks are combined in the following iteration until the best solution is reached. Another impactful study in this area of research was carried out by Chaudhary et al. [88] in 2017, who used “-omics” data from different platforms to predict survival classes in hepatocellular carcinoma. Their model stratified patients into distinct risk groups and demonstrated comparable performance to models that additionally used clinical data, such as gender, cancer grade, and other risk factors. Furthermore, relations between survival and mutations in *TP53*, high expression of *BIRC5*, and other types of genomic alterations were shown as well. Elmarakeby et al. [89] in 2021 discovered that alterations of formerly unrelated genes such as *MDM4*, *FGFR1*, or *MALM3* are associated with prostate cancer outcomes. For this they used a neural network with specific constraints: nodes represent a biological entity and edges their relations. By doing so, they limited the degree of connectivity in the network to incorporate prior biological knowledge and to restrict the computational complexity. The advantage of genomics in prognosis predictions lies in the ability to obtain data at multiple levels, which can range from genomic properties to its specific sequences. As a result, subtle changes in the cellular machinery can be identified as potential biomarkers. Nevertheless, compared to histopathology, many genomic biomarkers first need to be validated clinically to be translated into medical workflows.

An aspect that distinguishes AI in clinical genomics from histopathology is the diversity of model types used. Whereas in DL for histopathology basic model architectures were adapted from computer vision, DL in genomics did not find a direct analog in computer science, leading to a broader experimentation with various model types. For example, Chaudhary et al. [88] utilized an autoencoder, a form of DL, to integrate diverse omics data and then stratified liver cancer patients into risk groups. Yousefi et al. [90] deployed multi-layer perceptrons combined with a Cox survival model for prognosis predictions. Furthermore, random forests, gradient boosting, convolutional or graph-based networks, and more simple regression methods are applied in the field as well [91–94]. Today, similar to histopathology, transformer neural networks are becoming more and more prevalent in the field [95]. Taking into account the heterogeneity of genomic data, there is no single method that can be universally applied, underlining the need for continuous exploration in the future.

Cancer genomics remains a promising area for the application of DL. Many of the designated studies have shown to effectively complement bioinformatics tools and explore applications beyond them. Nevertheless, to our knowledge, DL tools for genomics have not yet received regulatory approval for clinical use. However, the cost for sequencing has dramatically decreased since the first human genome project, which indicates that genomic testing will probably become available to a broad range of cancer patients in the future [96, 97]. Therefore, we anticipate that DL in precision oncology will also benefit from more widely available genomic data. Apart from the application classes we mention in this review, DL could play numerous roles in clinical genomics in oncology. For example, DL could leverage tasks ranging from fundamental steps such as quality control or alignment to the high-level understanding of tumor evolution and timewise changes occurring in our genome. Finally, in routine clinical practice, DL could also be instrumental for screening purposes, such as in liquid biopsies for early cancer detection and disease monitoring.

## Multimodality

Gathering extensive information prior to making decisions is not an exclusive trait of AI. This is also common within clinical workflows, where physicians rely on a range of data, such as basic patient information, medical records, and test results, to inform their decisions. For these reasons, the field of multimodal AI has emerged in recent years, where the inputs of the models originate from various data sources and output a single prediction. A few studies have investigated data fusion from histopathology and genomics data, capitalizing on potential synergies between these data modalities, ultimately aimed at clinical use. Histopathology images are widely available and inexpensive, but only show tissue phenotype, not necessarily underlying molecular changes. Therefore, it was shown that already the addition of clinical parameters from the patient could improve the generalizability of DL models improving the predictions [21]. Genomic methods, on the other hand, can offer a glimpse into the underlying machinery within the cells, but there is still the disadvantage that a certain amount of material is required to obtain such information, which is not always feasible. Furthermore, technical aspects also need to be considered, as in the case of DL, where the model’s performance is critically dependent on the size of the input. Hence, the integration of data from different modalities could potentially allow for an increase in the information given to a model. With this, previously missing information can be completed or extended, refining the model’s predictions and subsequently improving biomarkers [15, 98].

One of the first to publish a multimodal DL model combining histopathology and genomics was Mobadersany et al. [99] in 2018. They combined WSIs, *IDH* mutation, and 1p/19q codeletion status data as input of a ML model to predict survival for patients with gliomas (Fig. 2a). Furthermore, their method surpassed several clinical biomarkers for prognosis. One year later, Cheerla and Gevaert [100] utilized RNA expression data in combination with WSIs for 20 cancer types in order to improve survival predictions. The most recent evidence indicating that utilizing multiple modalities can be superior to single modalities was provided by Chen et al., who published two separate models: PathomicFusion (2019), which integrated WSIs, driver mutation, copy number variation, as well as RNA-sequencing data, and PORPOISE (2022), which added genomic profiles to WSIs [17, 101]. In terms of performance, PathomicFusion was able to reach a c-index of 0.826 in glioma and 0.72 in clear cell renal cell carcinoma survival prediction. In PORPOISE, the best performance was achieved in kidney renal clear cell carcinomas with a c-index of 0.827. However, external validation of these results might be needed before clinically translating these models [102]. In addition to prognostication, other application types such as grading and subtyping were studied with multimodal models as well. Especially in brain cancer, many studies were carried out. For example, Pei et al. [103] predicted grading in gliomas based on the same features of Mobadersany et al. previously mentioned. This focus on brain cancer is likely due to the change in classification standards of gliomas in 2016, in which the World Health Organization added molecular features as decision standards to histopathological ones [104]. Thus, studies that would have solely relied on histopathology in the past, would now also require genomic evidence. In this way, clinical guidelines could facilitate multimodal research as well.

Adding another layer of multimodality, Boehm et al. [105] and Vanguri et al. [106] not only utilized histologic and genomic data but also expanded this repertoire by radiology images. With this, a next step towards a holistic integration of all clinically available information was taken, even though the complexity of these models would make their training and clinical deployment more difficult than single-modality models. Nevertheless, in a medical setting, having separate models for each data type will probably not be practical. Furthermore, in the future, it is possible that AI models not only incorporate patient data but also general medical information to make knowledge-based predictions. This could make them a universally applicable tool which combines predictions with practical reasoning that humans could interact with [107].

## Outlook

As a result of technical advancements over the past years, DL models are continually becoming more powerful and generalizable. Given enough data and a clearly defined task, DL models can in principle outperform human observers in patient diagnosis and potentially in downstream decision-making processes [108, 109]. Nevertheless, some key limitations need to be overcome when applying DL to precision medicine [110].

In ML, models require sufficiently large amounts of data to become good at their task. Part of this requirement is for technical reasons, as many repetitions of patterns are required to force the internal model parameters into their desired state. Another reason for data requirements, however, is the variability that is present in any biological system. In particular, tumors are diverse as their genotype, phenotype, and clinical behavior differ between patients. The minimum size of any training data set is such that it can represent the biological variability. Therefore, studies which only contain a dozen participants, will usually not have sufficiently diverse data to generalize well to external datasets, particularly in clinical routine [111]. In consequence, to make DL models available for a wide range of clinical settings, ever larger datasets need to be acquired and shared (Fig. 2b). Data collection, not model flexibility, is the main bottleneck in training DL solutions in cancer research and oncology. Histopathology, as the base of diagnosis, is more readily obtainable than genomic data, which is typically costly and not routinely acquired for all patients. Consequently, genomic cohorts are harder to establish, particularly for multi-omic approaches. Extensive clinical setups and infrastructure are required, often limiting them to ​well-funded research centers or large healthcare institutions. One way to address these challenges is through distributed learning such as federated or swarm learning, where peers that are prohibited from public data sharing can still jointly train models [112–114] (Fig. 2b). Furthermore, technical concepts could supplement data acquisition. Methods such as class balancing or augmenting datasets with simulated samples could aid studies with small patient numbers [115–117]. On the other hand, improved ML models could be more data-efficient and be able to sufficiently learn from even smaller datasets, potentially improving the data availability problem with a different strategy [118, 119].

In addition to limitations in dataset size, another fundamental problem of the development and deployment of DL systems in healthcare is that many datasets contain an internal bias based on the ethnicity, sex, or socio-economic circumstances of participants, or the institution in which the studies were conducted [120–122]. Consequently, this calls for fairer and more diverse data acquisition strategies for upcoming studies which, in reverse, would have a positive impact on the generalizability of DL models again (Fig. 2b). In addition, even in homogenous data, standards for data curation need to be established nationally and internationally to make data comparable between institutions in the first place (Fig. 2b). Furthermore, since changes can occur within populations AI is used upon, we will encounter the necessity for model updates and reconfigurations, a property mostly not considered in model design today (Fig. 2b) [123]. This will eventually allow obtaining DL models that dynamically learn during deployment, rather than being “frozen” after a single static training step.

Ultimately, the aim of the research presented in this review is to implement DL in actual clinical routines. Unfortunately, this is notoriously challenging, as most countries mandate a necessary but highly complex regulatory approval. Obtaining such regulatory approval is not attainable for academic teams, only for commercial enterprises with quality-controlled development workflows and the financial means to bring an algorithm to the market as a product [124]. Even after gaining approval, there are other additional challenges to overcome. For instance, few healthcare institutions even in the most economically prosperous countries are fully digitalized. Particularly, histopathology is based on the manual handling of glass slides in the overwhelming majority of healthcare institutions in the US and the EU today [110] (Fig. 2b). Moreover, a new skillset in healthcare providers and technical assistants is also needed to ensure processes are running efficiently. In the future, substantial investments are required to make healthcare infrastructure ready for a routine deployment of DL-based biomarkers (Fig. 2b).

Finally, for DL to be adopted by practitioners, the models should ideally not be considered as a "black box", but also inherit the explainability for their decisions (Fig. 2b) [125]. This challenge is difficult to address since DL models exhibit a high degree of complexity and are often susceptible to minor changes in the input data, making it difficult to ensure reliable and consistent outputs [126]. A number of established techniques exist which are often used to make models explainable. For histopathology, these include mostly two types: “saliency maps,” which highlight parts of the input data that were relevant for decision-making, and “extreme examples,” i.e., extracting the instances in the dataset that are assigned the highest and lowest prediction scores by the model [127]. In clinical genomics, particularly for tabular data, explainability methods such as Local Interpretable Model-agnostic Explanations (LIME) [128] or SHapley Additive exPlanations (SHAP) [129] values can indicate to which extent features influence predictions. However, the benefit of these methods depends on the human interpretability of the features themselves [130]. Furthermore, these approaches do not necessarily infer causality which shows that we are only at the beginning of this development. In addition to the explainability of specific models, generative AI could change the way we perceive what DL actually learns by reversing the DL workflow, creating data from an input query (Fig. 2b) [131]. More importantly, generative DL models could allow us to integrate counterfactuality. Essentially, as a first step, large DL models gather large and diverse knowledge about biological processes. Then, in counterfactual methods, the generative DL part can be used by a human experimentalist to answer questions such as “what would this particular tumor look like if it had a *BRAF* mutation?”, or “what would this precise tumor look like if the lymphocytes were removed?” [132, 133]. These approaches are not widely investigated in the analysis of pathology images or genomic data of cancer, but could be a useful tool for educational purposes and search for yet unknown properties.

In conclusion, the incorporation of AI into patient care is a multifaceted endeavor that requires extensive collaboration of researchers, healthcare institutions, and administrative bodies. The strategies explored in this review have the potential to enhance personalized treatments and advance precision oncology, possibly yielding cost savings and improved outcomes for patients. The rapid evolution of DL is remarkable, especially considering that just a decade ago it had virtually no role in the analysis of clinical data at all. Therefore, we anticipate that DL will become a widely used component of clinical workflows in precision oncology.

## Data Availability

Not applicable

## Abbreviations

AI: Artificial intelligence
AUROC: Area under the receiver operating characteristic
CNN: Convolutional neural network
FDA: Food and drug administration
H&E: Hematoxylin and eosin
ML: Machine learning
qPCR: Quantitative polymerase chain reaction
SVM: Support vector machine
TCGA: The Cancer Genome Atlas
WHO: World Health Organization
WSI: Whole slide image
